# Asynchronous Curriculum “Socially Synchronized”: Learning Via Competition

**DOI:** 10.5811/westjem.2018.10.39829

**Published:** 2018-11-30

**Authors:** Jon Smart, Adriana Segura Olson, Andrew Muck

**Affiliations:** The University of Texas Health Science Center-San Antonio, Department of Emergency Medicine, San Antonio, Texas

## Abstract

**Introduction:**

Now widespread in emergency medicine (EM) residency programs, asynchronous curriculum (AC) moves education outside of classic classrooms. Our program’s prior AC had residents learning in isolation, achieving completion via quizzes before advancing without the benefit of deliberate knowledge reinforcement. We sought to increase engagement and spaced repetition by creating a social AC using gamification.

**Methods:**

We created a website featuring monthly options from textbooks and open-access medical education. Residents selected four hours of material, and then submitted learning points. Using these learning points, trivia competitions were created. Residents competed in teams as “houses” during didactic conference, allowing for spaced repetition. Residents who were late in completing AC assignments caused their “house” to lose points, thus encouraging timely completion.

**Results:**

Completion rates prior to deadline are now >95% compared to ~30% before intervention. Surveys show increased AC enjoyment with residents deeming it more valuable clinically and for EM board preparation.

**Conclusion:**

Socially synchronized AC offers a previously undescribed method of increasing resident engagement via gamification.

## BACKGROUND

Widespread in emergency medicine (EM) residencies, asynchronous curriculum (AC) moves education outside the classic classroom setting, with the majority of current residents choosing to use various forms of AC.^1^ Previous studies suggest this model of learning to be non-inferior in comparison to traditional didactic education.^2^,^3^ Our EM residency’s prior AC had residents learn in isolation, achieving marks of completion via quizzes before advancing without the benefit of deliberate knowledge reinforcement or discussion with peers. Residents endorsed low levels of enjoyment using this format as well as low confidence in the AC improving their readiness for clinical work or EM board exams. We sought to increase resident engagement and spaced repetition by creating a social AC using gamification.

## OBJECTIVES

Our objectives were to increase resident engagement using gamification as well as to encourage spaced repetition. We measured these objectives by resident completion percentage, and by the residents’ subjective enjoyment of the new AC and their self-assessment of clinical and board exam preparedness.

## CURRICULAR DESIGN

We created a free, open-access website, AlamoCityEM.com, with a variety of monthly options consisting of free open-access medical education (FOAMed) resources and EM textbook chapters based upon our curriculum, broken up into one-month “blocks.” Resources were selected based upon an extensive search of available FOAMed performed by a post-graduate year-2 resident using search terms from key topics in the assigned textbook chapters for that month’s block. The resident’s search included a custom Google search engine created specifically for this project, which encompassed over 50 unique FOAMed websites and podcasts. These options were then evaluated by a faculty advisor for content suitability prior to being made available to the residency.

Each option was given an estimated time for completion. Time estimates for audiovisual options such as podcasts and videos were based upon actual run time at 1x speed. We input text from the textbook and article options into a website designed to estimate reading time, with resulting time estimates rounded upward to adjust for comprehension time.^4^ Residents self-selected a total of four hours’ worth of material each month, allowing for adjustment of choice length based on individual resident preferences.

In an innovative step, rather than using pre-created quizzes we asked residents to choose 12 learning points from the material that they deemed valuable. These learning points were submitted to a group Google spreadsheet to count as their mark of completion for the month. By identifying and submitting these learning points, learners were forced to consciously identify what they viewed as most valuable in the material they had covered. To further benefit from this process, residents were able to view learning points from their co-residents who often chose different resources each month. Thus, each month a crowdsourced document of 360 learning points was formed, allowing residents to benefit from each other’s asynchronous learning that had previously been performed in isolation.

An issue with our residency’s original AC was that once information was learned, there was no deliberate effort to reinforce that knowledge. This was particularly concerning as prior educational literature has shown that “spaced repetition” may be used to encourage knowledge retention.^6^ In another innovative step, we used the learning points from all residents to create monthly trivia competitions that were held during didactic conference. Divided into three “houses” (Sherlock, MacGyver, and Hawkeye), residents competed in teams to encourage social engagement and learning via gamification. These competitions have helped to break up traditional didactic learning in grand rounds by offering an interactive learning format that has included diverse styles to keep the competitions fresh and engaging.

Residents late to finish their asynchronous task prior to deadline caused their entire “house” to lose points for that month’s competition. Similarly, the first “house” with all members achieving completion received bonus points, giving them a head start in the competition. This camaraderie served to create a social expectation to finish in a timely manner. The overall competition spanned the academic year with the “house” winning the most months being declared the victors with prizes awarded.

## IMPACT/EFFECTIVENESS

After implementation of this previously undescribed AC model, our residents achieved completion rates of ~ 95% prior to deadline compared to ~30% preceding intervention. Particularly useful to faculty was encouragement of timely completion via penalty and bonus points. On anonymous, standardized Likert surveys, residents reported markedly increased enjoyment of the curriculum and ranked it as subjectively more valuable for improvement in both clinical practice and board preparation (Figure). Strengths noted by residents included the range of options, variety of choice in time lengths, and the “house” system developed for competitions. Junior residents particularly found it useful to see learning points submitted by senior residents. A specific area for improvement noted by residents was a request for a more streamlined format than Google spreadsheets for submission of their learning points.

The monthly trivia competitions were a well-received deviation from standard didactic lecture during conference. To date we have trialed 10 unique trivia formats with the highest rated formats being those that encouraged discussion between residents prior to answer submission as opposed to formats requiring immediate answer submission. These competitions have been made available as FOAMed on the AlamoCityEM.com website so that residents unable to attend that month may use them, as may learners not associated with our institution. One limitation of the study was that we measured subjective rather than objective outcomes. Future possible directions of study include assessing the effect of the new AC model on in-service examination scores as well as long-term knowledge retention. Another potential area of improvement might be the creation of a standardized method for faculty to evaluate the included asynchronous sources. Another fruitful area for future research would be to examine the trends in residents’ preferences of both length and media format.

## CONCLUSION

Overall, this socially synchronized AC model offers a previously undescribed method of encouraging resident engagement and spaced repetition. The “socially synchronized asynchronous” model may be easily adapted by other residencies with minimal start-up effort, requiring only creation of spreadsheets unique to their own learners and approximately one hour of didactic time monthly for trivia competitions.

## Figures and Tables

**Figure:**
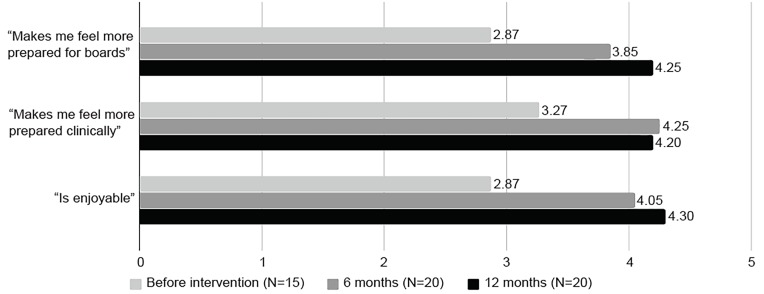
Resident response to intervention based on standardized Likert scale.
